# Primary and Secondary Sinonasal Aspergillosis in Dogs

**DOI:** 10.3390/ani15192880

**Published:** 2025-10-01

**Authors:** Sarah Rösch, Gerhard Ulrich Oechtering

**Affiliations:** Ear, Nose, and Throat Unit, Small Animal Department, College of Veterinary Medicine, Leipzig University, 04102 Leipzig, Germany

**Keywords:** interventional endoscopy, mycotic rhinitis, nasal debridement, nasal discharge, rhinoscopy, sinoscopy

## Abstract

**Simple Summary:**

Canine sinonasal aspergillosis (SNA) is a fungal infection caused by *Aspergillus* species, affecting the nasal cavity and possibly the paranasal sinuses. Most studies have focused on dogs with SNA that do not have concurrent nasal pathology (singular SNA), aiming at clarifying its poorly understood etiopathogenesis, therapy, and prognosis. In contrast, little is known about treatment outcomes in dogs with SNA and concurrent nasal pathology or in cases where SNA is confined to the nasal cavity without sinus involvement. We hypothesized that this form of SNA may be more common in dogs with both SNA and an additional nasal disorder. We retrospectively reviewed medical records of 30 dogs diagnosed with SNA through a comprehensive workup including cross-sectional imaging and rhinoscopy for initial diagnosis and follow-up. Our findings demonstrate that computed tomography (CT) and rhinoscopy are essential to detect co-existing nasal diseases. Moreover, dogs with either additional disease or localized nasal singular SNA showed better treatment outcomes and often required fewer follow-up visits. In some cases, complete resolution was achieved after a single procedure. These results are highly relevant for clinical practice, as they emphasize the importance of thorough diagnostics, help guide treatment decisions, and improve communication with pet owners regarding prognosis.

**Abstract:**

**Introduction:** Canine sinonasal aspergillosis (SNA) can present singular as a primary disease or secondary to concurrent sinonasal pathology. We hypothesized that treatment response and prognosis differ between both forms, particularly when sinusitis is present. **Methods:** In this retrospective study, 30 dogs with SNA were categorized as either group pA (primary aspergillosis) or group sA (secondary aspergillosis; with additional sinonasal pathology). History, diagnostics, endoscopic therapeutic intervention of affected nose and sinus, and follow-up data were analyzed. **Results:** Group pA included 19/30 dogs (63%), with 15 dogs (79%) showing concurrent sinusitis. Group sA included 11/30 dogs (37%; additional conditions: foreign bodies, dental pathologies, frontal bone fracture). Only 2/11 sA dogs (18%) had sinusitis. Follow-ups in group pA were more frequent than in group sA (*p* = 0.04). Need for re-treatments differed significantly between groups (*p* = 0.02) and in dogs with sinusitis, regardless of group (*p* < 0.001). In group sA, treating the underlying condition plus single endoscopic debridement ± antifungal therapy led to clinical resolution in 11 of 12 dogs (92%). **Conclusions:** Primary SNA is frequently associated with sinusitis, requires aggressive repeated antifungal therapy, and may not achieve a definitive cure. Secondary SNA is usually confined to the nasal cavity, responds well to underlying condition treatment, carries better prognosis, and requires fewer antifungal treatments.

## 1. Introduction

Canine sinonasal aspergillosis (SNA) is characterized by a unilateral or bilateral infection of the frontal sinus and nasal cavity with *Aspergillus* spp. [1]. In dogs, *Aspergillus fumigatus* is the most identified species [2]. Although it is a relatively rare disease in dogs, it is a common cause of chronic nasal discharge, ranging from 7% to 34% [3,4,5]. In humans, infections with *Aspergillus* spp. are reported in healthy as well as in immunosuppressed patients [6], whereas in dogs, animals typically show no evidence of systemic immunodeficiency [1,7,8]. It is hypothesized that local deficiencies in mucosal defenses predispose to fungal infection [1,9]. The disease is thought to originate in the frontal sinus and subsequently spread to the nasal cavity [10]. However, some studies report cases where the frontal sinus is not involved and fungal granulomas are confined to the nasal cavity alone [10,11]. Reported alterations to the frontal sinus during aspergillus infection include fluid accumulation, mycotic granuloma, and bone alterations such as lysis or proliferation [10]. Various treatment regimens, ranging from minimally invasive to surgical approaches, have been described [12,13,14]. In general, treatment can be prolonged and challenging, and a definitive cure is not always achieved [12,13,14]. Even after treatment, dogs may continue to exhibit signs consistent with chronic rhinosinusitis [15,16]. In addition to typical SNA presentations, some dogs with aspergillus infections occasionally present with coexisting sinonasal pathologies, such as plant foreign bodies [10,11,17].

Building upon previous studies and our own clinical experience in the treatment of dogs with SNA, we hypothesized that dogs with aspergillosis and concurrent nasal or perinasal pathology and without sinusitis are easier to treat and have a better prognosis. This contrasts with dogs suffering from SNA alone, typically without any additional nasal disease, but frequently with accompanying sinusitis.

To assess this hypothesis, we defined the following aims:

1. Characterization of dogs with SNA: Evaluation of data from cross-sectional imaging, diagnostic video rhinoscopy, histologic examination of mucosal biopsies, and mycologic examination of mucosal swabs, both obtained under endoscopic guidance.
2. Classification of dogs into two groups:
  - Group pA (primary aspergillosis), including dogs with SNA without additional nasal or perinasal findings.
  - Group sA (secondary aspergillosis), including dogs with SNA and an additional nasal or perinasal pathology.
3. Comparative evaluation of the therapy and the course of the disease in dogs of both groups:
  - Treatment of the underlying disease for dogs of group sA;
  - Endoscopic therapeutic intervention of aspergillosis with debridement and antifungal topical medication for dogs of both groups;
  - Follow-up examinations with computed tomography (CT), video-endoscopic examination of the nose and paranasal sinuses, laboratory tests, and telephone consultations with the owners;
  - If necessary, repeated endonasal interventional therapy, e.g., performed similarly to the first treatment.

## 2. Materials and Methods

### 2.1. Selection Criteria and Assignment to Groups

Medical records of dogs with diagnosis of SNA between 2013 and 2019 were identified by the authors in the data set of the Ear, Nose, and Throat (ENT) unit of the Small Animal Department of Leipzig University. Diagnosis of SNA was based on at least two positive diagnostic findings indicating aspergillus infection, including rhinoscopy, CT, and laboratory tests [18]. For each case in this group of SNA dogs, the following information was obtained if available: patient demographics, medical history, clinical findings, CT, exploratory sino-rhinoscopy, and laboratory findings (e.g., fungal culture and histopathology). CT-scans and complete video recordings of exploratory and interventional sino-rhinoscopies were independently but not blinded re-evaluated by the authors.

All patients underwent therapeutic intervention via interventional sino-rhinoscopy. The method of intervention, extent of topical treatments, and postoperative medication were evaluated. Follow-up examinations included repeated diagnostics (CT, endoscopy, and others) and, when indicated, repeated therapy and intervention. The decision for repeated treatment was at the discretion of the treating veterinarian, who was an experienced clinician specialized in ENT disorders and diagnostic evaluations.

Based on a retrospective review of the diagnostic findings (CT and rhinoscopy), dogs were assigned to one of two groups:

- Group pA: SNA dogs without additional nasal or perinasal pathology, primary aspergillosis.
- Group sA: SNA dogs with additional nasal or perinasal pathology, secondary aspergillosis. Concurrent pathologies were defined as clearly identifiable and well-documented pre-existing conditions such as dental disease (based on CT images) or foreign bodies (identified via CT and/or endoscopic evaluation). These findings, e.g., plant-based nasal foreign bodies or periapical dental pathology, were objectively evident on imaging, confirmed by experienced clinicians, and documented at the time of diagnosis and treatment. Therefore, an unblinded re-evaluation of the imaging data was deemed adequate for the purposes of this study.

### 2.2. Ethical Approval

The study was conducted in accordance with the guidelines of the Leipzig University Ethics Committee and approved under protocol number EK15/2025. All animals received treatment based on their individual medical needs and according to the best clinical judgment of the attending veterinarians. Written informed consent was obtained from the owners of all animals involved in the study for the use of clinical data and diagnostic samples.

### 2.3. Diagnostic Procedures

#### 2.3.1. Anesthesia

Dogs were anesthetized for CT, rhinoscopy, and therapeutic intervention. A standardized anesthetic protocol with minimal variation was used, adapted to the individual requirements of each patient and at the discretion of the attending anesthetist [19].

#### 2.3.2. Cross-Sectional Imaging

**Figure 1:**
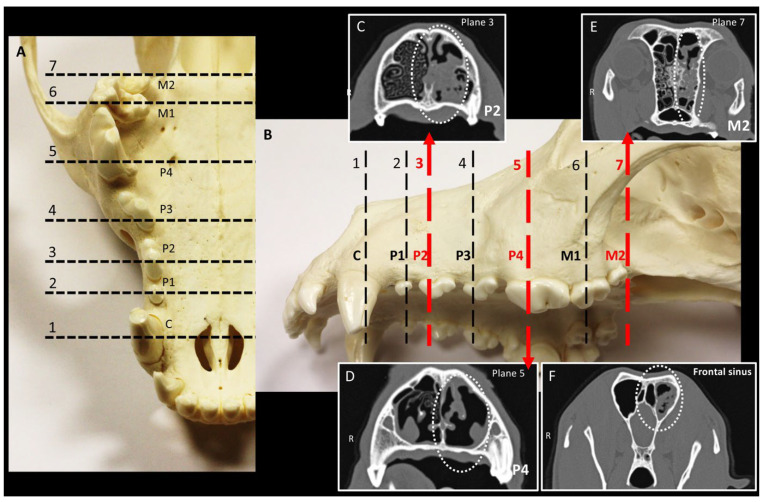
**Landmarks for comparative evaluation of sinonasal computed tomography (CT) findings.** (**A**,**B**) The skull of a dog; section planes 1 to 7 are oriented to the teeth of the maxillary jaw. (**C**–**F**) Transverse CT images from a dog with primary sinonasal aspergillosis in the left nasal cavity with sinus involvement, (**C**) at the level of the maxillary second premolar (P2; Triadan 106/206), (**D**) maxillary fourth premolar (P4; Triadan 108/208), and (**E**) maxillary second molar (M2; Triadan 110/210), respectively. (**F**) Sectional plane at the level of the caudal end of the sphenoid sinus.

Dogs were positioned in the sternal recumbent position with the hard palate fixed parallel to the scanning table. Transverse image series (1.0 mm) were acquired and used for reformation to obtain sagittal and dorsal views. During CT follow-up studies, efforts were made to ensure comparable positioning of the dog in all examinations, data were analyzed in the same way, and the time between follow-up examinations was documented. For comparison of sectional planes on CT at initial and follow-up scans, the changed nasal cavity areas were determined based on the maxillary teeth in the transverse section. The image series was adapted to a ww/wl 3000/300 for this purpose. In each case, the tooth at the longitudinal center of the transverse section was selected as the reference point (largest size of the pulp cavity; see Figure 1). For the two-rooted maxillary second premolar (P2; Triadan 106/206) and maxillary third premolar (P3; Triadan 107/207), the rostral root was used as the reference point. For the three-rooted maxillary fourth premolar (P4; Triadan 108/208) and maxillary first molar (M1; Triadan 109/209), the rostral lingual and medial roots were considered, as both roots lay in the same sectional plane when properly aligned. For the maxillary secondary molar (M2; Triadan 110/210), the caudal border of the tooth was used as the reference. The frontal sinuses were evaluated at the level of the caudal end of the sphenoid sinus (see Figure 1).

#### 2.3.3. Diagnostic Rhinoscopy

The upper airways of all animals were examined according to a standard endoscopy protocol (Table 1) [19]. All endoscopies were recorded in full high-definition (FHD) video quality (H3 Spiess high-definition camera head, TH100, Karl Storz, Tuttlingen, Germany) and digitally archived (with the AIDA system, Release 1.3, 100–240 VAC, 50–60 Hz, Karl Storz, Tuttlingen, Germany).

**Figure 2:**
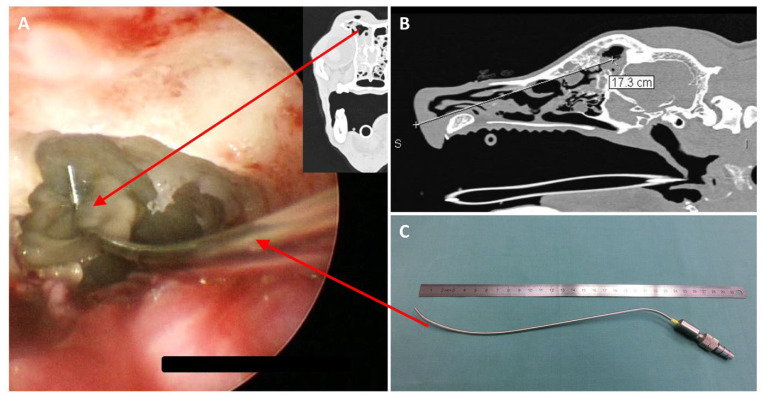
**Sinoscopy in a dog with primary sinonasal aspergillosis (SNA).** (**A**) Endoscopic view into the frontal sinus during debridement using a curved suction tube. (**B**) Sagittal computed tomography (CT) image of a dog’s skull showing sinusitis; a linear measurement from the nasal entrance to the frontal sinus is indicated. (**C**) Curved suction tube next to a ruler for size reference.

The dogs were placed in sternal recumbency with maxillary fixation caudal to the canines; the mandible was left unrestrained. The following endoscopically obtained findings were documented: changes in the nasal mucosa of the turbinates and nasal septum, fluid accumulation, localization of mycotic plaques or granulomas, a pathologically open entrance to the frontal sinus, and evidence of a foreign body or other pathology. A mycotic plaque or granuloma was characterized by a firm, yellowish-white mass with a mold-like appearance on the surface (Figure 2).

#### 2.3.4. Posterior Rhinoscopy

The oro- and laryngopharynx were examined prior to post-rhinoscopic examination of the nasopharynx. Secretions draining from the nasopharynx were identified and suctioned if necessary. The nasopharynx was subsequently visualized using a 120° rigid endoscope (Table 1). Visible structures included the openings of the *Eustachian tubes*, the *tonsilla pharyngea*, the caudal border of the nasal septum, and the lumina of both *meatus nasopharyngei* [19].

#### 2.3.5. Anterior Rhinoscopy

Rhinoscopy began with visualization of the nasal plane and the nostrils. Secretion residues, swellings, or depigmentation were documented. Rigid endoscopy of the nasal cavity always began on the unaffected or less affected nasal cavity. Initially, no irrigation was performed; secretions were first aspirated with a suction tube that was introduced parallel to the endoscope. The endoscope was passed through the nasal vestibule, medially passing the large *bulbus nasi*, the end of the alar fold. In the healthy nose, the 5-fold view would provide medial the view on the nasal septum with ventral and dorsal septal swell bodies and lateral on the *plica recta*, *plica alaris*, and *plica basalis*. The transition of the *plica alaris* into the dorsal and ventral lamellae of the *concha nasalis ventralis* followed. Caudal to the ventral turbinate, there was the nasal exit below the wing of the *vomer*. The endoscope was further passed through the nasopharyngeal meatus to assess the nasopharynx with *tonsilla pharyngea* and the openings of the auditory tube.

#### 2.3.6. Sinoscopy

In dogs with SNA affecting the frontal sinus, the sinus is often readily accessible endoscopically due to the pathologically widened opening. In contrast, in healthy dogs, endoscopic visualization of the frontal sinus is generally not possible because of the narrow anatomical connection between the nasal cavity and the frontal sinus [20]. Notably, a markedly widened ostium is not consistently present in all cases of mycotic sinusitis [11].

As a result, evaluation of the frontal sinus was initially performed using the cross-sectional images in the included dogs with aspergillosis. During subsequent rhinoscopy, the frontal sinus was assessed for content and whether the opening to the frontal sinus had already been widened due to the aspergillus infection. If alterations to the frontal sinus were detected in the cross-sectional images (e.g., fluid accumulation or bony alterations to the frontal bone), but no pathological widening was observed during rhinoscopy, endoscopic therapeutic intervention to open the frontal sinus was performed (Figure 2).

#### 2.3.7. Laboratory Tests

Culture-dependent mycological and bacteriological examinations were performed on nasal swabs collected sterile from deep within the nasal passages (IDEXX Laboratories, Kornwestheim, Germany). Biopsies of the nasal mucosa and/or granuloma tissue were obtained in a standardized manner under endoscopic guidance for histopathological examination [21]. Histopathological examinations were conducted by veterinary pathologists at Antech Lab Germany GmbH, Tierpathologie Munich (Munich, Germany, with Dr. W. von Bomhard, Dipl. ECVP).

### 2.4. Therapy

#### 2.4.1. Endoscopic Therapeutic Intervention

**Figure 3:**
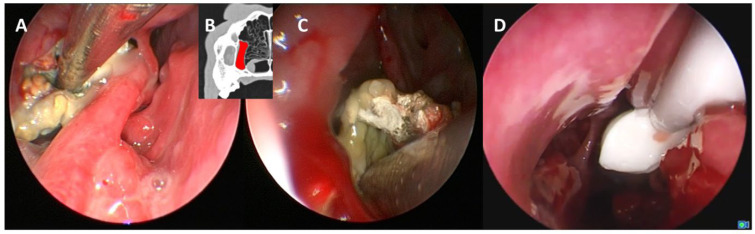
**Interventional endoscopy: debridement of the maxillary recess and instillation of antifungal ointment.** (**A**) Debridement of the maxillary recess using a rigid suction tube. (**B**) Computed tomography (CT) image highlighting the maxillary recess (marked in red). (**C**) Endoscopic mobilization of a fungal plaque. (**D**) Instillation of antifungal ointment into the maxillary recess.

Following diagnostic rhinoscopy, all animals underwent thorough endoscopic interventional debridement of the nasal cavity and paranasal sinuses via an endonasal approach. Initially, secretions were removed using various rigid and flexible suction tubes. Fungal granulomas were removed mechanically via suction or with forceps (Figure 3). Severely damaged or necrotic soft tissue was removed with a power vacuum. Necrotic bone and cartilage tissue (turbinate remnants) were then ablated using a combination of a power vacuum and various forceps. If involvement of the frontal sinus was identified, access was established using a septal punch. Complete debridement of the sinus lumen was then performed using curved instruments, including suction tubes, nibbling forceps, curved Kirschner wires, and pressure irrigation devices. Finally, the nasal cavity and the paranasal sinuses were thoroughly flushed with 0.9% saline (NaCl 0.9%, Braun, Melsungen, Germany) and a highly diluted povidone–iodine solution (Betaisodona solution, Mundipharma, Limburg, Germany; 1 mL Betaisodona solution per 100 mL NaCl).

#### 2.4.2. Topical Antifungal Therapy

**Figure 4:**
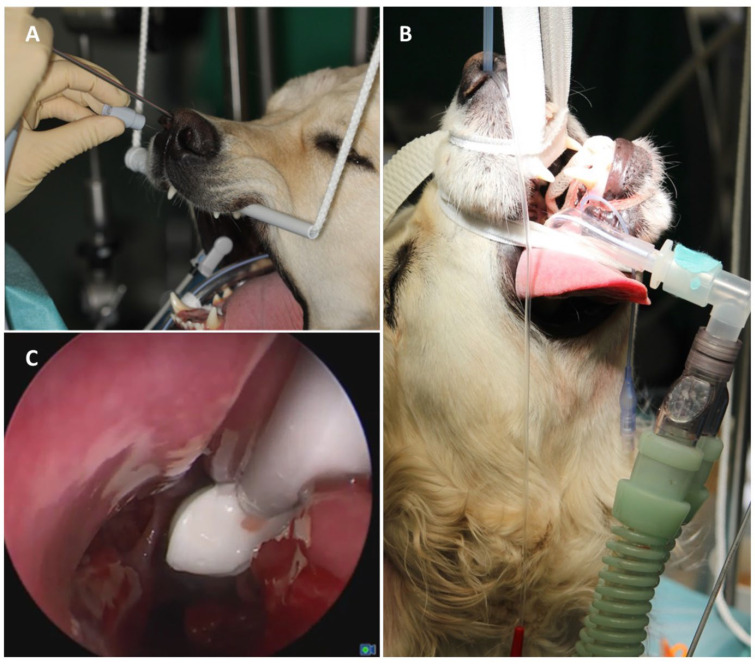
**Positioning of the canine patient for rhinoscopy and topical antifungal therapy.** (**A**) Bimanual endoscopic therapeutic intervention via the nasal entrance using a rigid endoscope. (**B**) Patient positioning and preparation: after sealing the nasopharynx, the nasal cavity and frontal sinus can be irrigated with an antifungal solution. (**C**) Instillation of antifungal ointment into the maxillary recess.

Antifungal therapy was performed using one of four different topical treatment regimens:

(1) Topical application of clotrimazole cream (c-cream; Canesten^®^, 1% clotrimazole, Bayer, Leverkusen, Germany, 3–7 mL per side depending on the size of the nasal cavity in different dogs and whether the frontal sinus was affected);
(2) Irrigation treatment with 1% clotrimazole solution (c-solution, Antifungol^®^, Hexal, Holzkirchen, Germany);
(3) Irrigation treatment with 1% enilconazole solution (e-solution; Imaverol^®^, Lilly Germany GmbH Elanco, Bad Homberg, Germany);
(4) C-cream deposition after irrigation treatment with c-solution or e-solution using a small plastic catheter endoscopically placed into the nasal cavity and region of interest (Figure 3 and Figure 4).

In general, as illustrated in Figure 4, after positioning the head in an upright position and sealing the nasopharynx, the nasal cavity and frontal sinus were completely filled with the antifungal solution, up to the level of the nasal planum, and they were maintained in this position for 30 min, with repeated endoscopic checks to ensure a constant fluid level. Due to the retrospective nature of the study and the potential need for repeated adjustments to achieve a tight seal, the exact volume of antifungal solution used per animal could not be determined retrospectively. The duration of treatment varied depending on the individual animal’s needs (such as anesthesia duration and any complications) and was based on the clinical judgment of the attending veterinarian. The procedure was well tolerated, with no intra- or post-procedural adverse events noted.

#### 2.4.3. Treatment at Home by the Owner

In dogs of group sA, post-interventional care included administration of a non-steroidal anti-inflammatory drug (NSAID, e.g., meloxicam [Metacam^®^, 1.5 mg/mL suspension, Boehringer Ingelheim, Ingelheim/Rhein, Germany; initial 0.2 mg/kg, followed by 0.1 mg/kg q 24 h]) for 1–5 days after debridement of the nasal cavity and/or dental treatment.

For dogs in group pA, similar post-treatment instructions included the administration of an NSAID for 5–10 days (see dosage above). Additionally, owners were instructed to provide mucolytic therapy with acetylcysteine (ACC 200 mg powder, acetylcysteine, Hexal, Holzkirchen, Germany) at a dose of 3–5 mg/kg orally, two to three times daily. The recent literature suggests that ACC has additional anti-inflammatory effects and promotes epithelial regeneration, as demonstrated in other animal species [22,23]. To support mucociliary clearance during wound healing and infection, the following measures were also recommended: once-daily inhalation with 0.9% saline solution for 15 min, or nasal flushing of the affected cavity with 2 mL of saline solution.

### 2.5. Follow-Up Examinations and Classification of Treatment Outcomes According to Need for Further Therapy and Intervention

At least one follow-up examination was recommended for each dog diagnosed with SNA, including CT, comprehensive rhinoscopy, histopathological examination of mucosal biopsies, and mycological examinations of nasal swabs. Follow-up was advised 4 to 8 weeks after treatment in any dog with SNA. Based on the findings, repeated or further treatments and additional follow-up examinations were required. The number of follow-ups and the need for repeated therapy were documented. Changes in antifungal therapy between the initial and follow-up treatments were made at the discretion of the ENT specialists (G.U.O. or S.R.) based on the animal’s disease status and progression.

Final treatment outcome classification was based on treatment decisions and interventions during follow-up examinations, as they reflect the success or failure of initial therapy. This classification provides a more objective evaluation of treatment outcomes, since the decision to administer or withhold antifungal therapy was based on: (1) clinical presentation (clinical signs yes/no, with structured owner interviews based on published questionnaires; more subjective observations of the owner), (2) diagnostic findings from re-examination under anesthesia (CT/rhinoscopy), and (3) results of additional diagnostic tests (e.g., histopathology, mycological examination). It should be noted that not all test results (e.g., histopathology, mycological examination) were available at the time of follow-up/rhinoscopy and that they may yield false-negative results.

Based on these criteria, in the follow-up examination, dogs were classified as follows:

(a) *NRTA* (no repeated antifungal therapy for aspergillosis); dogs are in complete remission:
  1. No clinical signs or nasal discharge were reported by the owners;
  2. Rhinoscopy showed no fungal granulomas or significant purulent secretions;
  3. All diagnostic test results were ultimately negative.
(b) *PTA* (preventive antifungal therapy for aspergillosis); antifungal treatment was performed as a preventive measure, at the discretion of the attending veterinarian:
  1. No clinical signs or nasal discharge up to maximally rarely/mild clinical signs were reported by the owners;
  2. Rhinoscopy revealed non-specific but eventually suspicious findings (e.g., increased nasal secretions, a subjectively slightly yellow discharge, or a more pronounced redness of the mucosa) but no evidence of fungal granulomas on endoscopy;
  3. Ultimately negative test results received.
(c) *RTA* (repeated antifungal therapy for aspergillosis); dogs are refractory and requiring repeated antifungal therapy:
  1. Showing ongoing clinical signs;
  2. Visible fungal growth in the rhinoscopic examination;
  3. And/or positive diagnostic test results for aspergillosis.

Additionally, two further groups were defined, reflecting real-world clinical scenarios, as some owners did not allow follow-up examinations under anesthesia:

(d) *“Zero follow-ups”:* Although strongly advised, at the owner’s request, no follow-up examinations were performed in dogs that showed no clinical signs in the long term (at least one year). Repeated owner interviews were conducted by an experienced ENT specialist. Even if clinical signs do not always accurately reflect the underlying condition in nasal cavity disease [24], it should be mentioned that these dogs stayed asymptomatic and were never presented for re-evaluation after the initial treatment. In addition to the owner’s subjective perception of whether the disease was still present or whether the stress and risk of examination under anesthesia were justified, follow-up examinations could also be influenced by factors such as financial constraints, concerns about anesthetic risk, and stress for the animal.
(e) *“Lost to follow-up”:* Dogs were considered lost to follow-up if no reliable information on disease status could be obtained and with therefore questionable clinical status.

### 2.6. Statistics

Statistical analyses were performed with GraphPad Prism (v10.4.2 GraphPad Software, La Jolla, CA, USA). Testing for normal distribution of values was performed using the D’Agostino and Pearson normality test. For the analysis of the parametric data, after testing for equality of variance by the Brown–Forsythe test and Bartlett’s test, one-way ANOVA was performed, followed by Bonferroni’s post hoc correction for alpha error accumulation. Results are given as mean ± standard deviation. For the analysis of the nonparametric data, the Mann–Whitney test was performed for less than three groups, and the Kruskal–Wallis test was performed for more than three groups, followed by Dunn’s multiple comparisons test. Results are expressed as median values with interquartile range (IQR). For evaluation of a nonrandom association between two categorical variables, such as the type of disease (primary versus secondary aspergillosis with and without frontal sinus involvement) and the relationship to the necessary treatment, Fisher’s exact test was used. A *p*-value < 0.05 was considered significant.

## 3. Results

### 3.1. Included Dogs and Assignment to Groups

Thirty dogs met the inclusion criteria. Nineteen of the thirty dogs (63%) showed no evidence of concurrent nasal or perinasal pathology and were assigned to group pA. In this group, SNA was diagnosed at the initial presentation in 15/19 dogs and at the second presentation in 4/19 dogs. In these four dogs, the imaging findings were suggestive of disease, but all initial diagnostic results, including rhinoscopy, histopathology, and mycological examination, were negative. Due to recurrent or persistent clinical signs of nasal cavity disease, the diagnosis of SNA was made in a follow-up examination: 10 months after the initial presentation in one dog, 4 months in another, and 1 month in two dogs. At this point, mycotic granulomas were detected in rhinoscopy (*n* = 4; histopathology positive in three dogs; mycological examination positive in two dogs), and the first treatment (debridement and antifungal therapy) was initiated.

**Figure 5:**
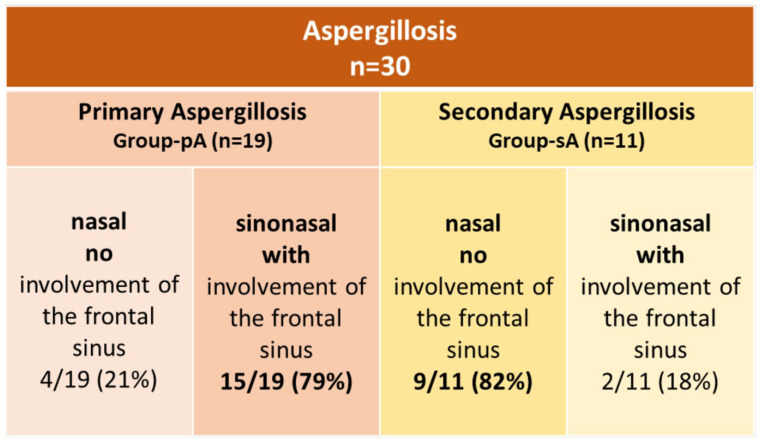
**Illustration of the dogs with sinonasal aspergillosis (SNA) included in this study, categorized as primary (pA) or secondary (sA) aspergillosis.** Dogs were divided into two groups based on the presence or absence of concurrent nasal or perinasal pathology and further subdivided according to the presence of frontal sinus involvement. Fisher’s exact test revealed that dogs with primary aspergillosis were significantly more likely to have frontal sinusitis than those with secondary aspergillosis (odds ratio = 16.88, 95% CI: 2.70–90.02, *p* = 0.002).

Frontal sinus involvement was present in 15/19 dogs in group pA (79%), while 4/19 dogs (21%) had nasal aspergillosis without sinusitis (Figure 5). Sinusitis was detected in 13/15 dogs during the initial presentation and in 2/15 dogs at the first follow-up: one with a mycotic granuloma and one with fluid accumulation (Figure 5).

Eleven of the thirty dogs (37%) presented with additional nasal or perinasal pathology and were assigned to group sA. A plant-based nasal foreign body was diagnosed in 6/11 dogs (55%), dental root pathology in 4/11 dogs (36%), and an impression fracture of the frontal bone in 1 dog (1/11; 9%). In 9/11 dogs in group sA (82%), aspergillus infection was confined to the nasal cavity, whereas in 2/11 sA dogs (18%), frontal sinus involvement was also identified (Figure 5).

### 3.2. Medical History, Signalment, and Clinical Findings

All 30 dogs were normocephalic [25]. Included dogs were of the following breeds (number of dogs in the sA group is given in square brackets): Labrador Retriever (6 [sA: 3]; 20%), Golden Retriever (4 [sA: 0]; 13%), Appenzeller (2 [sA: 1]; 7%), mixed breed (2 [sA: 2]; 7%), Rhodesian Ridgeback (2 [sA: 1]; 7%), German Shepherd mix (1 [sA: 1]), Goldendoodle (1 [sA: 0]), Poodle (1 [sA: 1]), Dachshund mix (1 [sA: 0]), Galgo (1 [sA: 0]), Rottweiler (1 [sA: 0]), Briard mongrel (1 [sA: 0]), Giant Schnauzer (1 [sA: 1]), Welsh Terrier (1 [sA: 1]), Medium Schnauzer (1 [sA: 0]), Schnauzer mongrel (1 [sA: 0]), Mountain dog mongrel (1 [sA: 0]), Hovawart (1 [sA: 0]), and Jack Russell (1 [sA: 0]). Therefore, in group pA (*n* = 19), 8/19 dogs (42%) belonged to the Retriever breeds (Labrador, Golden Retriever, and Golden Doodle).

A total of 12 [sA: 5] of the 30 dogs (40%) were male, 6 [sA: 2]/30 (20%) male castrated, 9 [sA: 3]/30 dogs (30%) were female, and 3 [sA: 1]/30 (10%) were female castrated. The mean weight was 29.0 ± 11.7 kg and was not significantly different between the two groups (data passed normality tests (alpha = 0.05), unpaired *t*–test, two-tailed, *p* = 0.4). Dogs in group pA had a mean weight of 30.4 ± 12.0 kg, and dogs in group sA had a mean weight of 26.6 ± 11.1 kg. The mean age of the included dogs at presentation to the ENT unit was 64.5 months [IQR 34.8–119]. A total of 5 [sA: 4] of the 30 dogs (17%) were younger than 2 years old at presentation, 15 [sA: 4]/30 dogs (50%) were 2–8 years old, and 10 [sA: 3]/30 dogs (33%) were older than 8 years. Therefore, four of the five dogs younger than 2 years of age belonged to group sA and had additional nasal or perinasal pathology. For the calculation of the duration of clinical signs before presentation to our clinic, one dog, for which the exact duration was only estimated at approximately 7 weeks and not precisely specified, was excluded. The duration of clinical signs of the other dogs (29/30) was 3 months (IQR: 1.5–6 months). A total of 9 of the 29 dogs (31%) showed clinical signs for more than half a year (>6 months), and 1 dog even showed signs for 44 months. The duration of signs was not significantly different between the dogs in group pA (3 months, IQR: 1.5–6 months) and the dogs in group sA with another nasal cavity pathology in addition to aspergillosis (3 months, IQR: 1–9 months; data did not pass normality tests (alpha = 0.05); Mann–Whitney’s test, two-tailed, *p* = 0.66, Hodges–Lehmann’s test 0.75 (95.06% CI: −2.0 to 5.0)). There was no significant difference in age, weight, or duration of clinical signs between group pA and group sA.

The observed clinical signs for pet owners were serous-to-purulent nasal discharge (30 [sA: 11]/30; 100%), epistaxis (17 [sA: 4]/30; 57%), sneezing (15 [sA: 4]/30; 50%), and reverse sneezing (3 [sA: 2]/30; 10%). In 20 [sA: 7]/30 dogs (67%), the quality of the discharge changed during the disease. Nasal discharge was observed by owners in 11/30 (37%) dogs on the right side, in 14/30 dogs (47%) on the left side, and in 5/30 dogs (16%) on both sides. No dog showed neurological signs. Only 4/30 dogs (13%, sA: 1 with dental pathology) showed pain during head manipulation. Of all dogs included, only one dog from group sA with a nasal foreign body had an elevated body temperature of 40.1 °C (3%).

A total of 29 [sA: 10]/30 (97%) dogs were pretreated with medication, 25 [sA: 9]/29 dogs (86%) were pretreated with one or more antibiotics, and 4 [sA: 2]/29 (14%) of these dogs were pretreated additionally with a corticosteroid. Twenty [sA: 8] of the twenty-five (80%) dogs showed no improvement after the administration of an antibiotic but rather a progression of nasal discharge. Three [sA: 0] of the twenty-five dogs (12%) showed worsening with bloody nasal discharge. Two [sA: 0] of the twenty-five dogs (8%) had an initial low-grade improvement for the owner, defined by a decrease in the amount of nasal discharge.

### 3.3. Results of Diagnostic Procedures

At initial diagnosis, a thorough endoscopic examination was performed in all 30 dogs (100%). Cross-sectional imaging was conducted in 29/30 dogs (97%), with CT performed in 25/29 dogs (86%) and MRI in 4/29 dogs (14%). Only one dog of group sA, which had a nasal foreign body, did not undergo cross-sectional imaging due to financial reasons.

A nasal mucosal biopsy was taken in 27/30 dogs (90%) for histopathological examination. Nasal cavity swabs were submitted for culture-based mycological examination in 24/30 dogs (80%) and for culture-based bacteriological examination in 19/30 dogs (63%). Positive results were obtained in 16/27 (59%) histopathological examinations and in 12/24 (50%) mycological cultures.

The diagnosis of SNA was made at initial presentation by CT and endoscopy in 26 [sA: 11]/30 dogs (87%). In the remaining 4 [sA: 0]/30 dogs (13%), the diagnosis was only made upon repeat CT and endoscopy, as all initial findings—including CT, endoscopy, mycological and histopathological examination—were negative for fungal infection.

**Figure 6:**
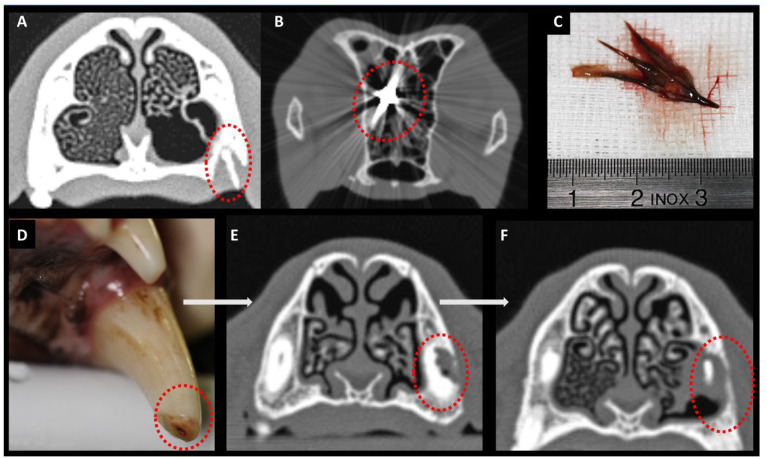
**Additional nasal diseases in dogs with secondary aspergillosis.** Dental root pathologies (**A**), metallic (**B**), and plant (**C**) foreign bodies cause secondary nasal cavity infection with *Aspergillus fumigatus*. (**D**) Clinical view of a maxillary canine tooth with an open pulp cavity. (**E**,**F**) Sequential cross-sectional computed tomography (CT) images (rostral to caudal) illustrating the extent of root damage (dotted circle). (**B**) Metallic foreign body at the level of the cribriform plate (dotted circle; bullet). This dog also showed secondary aspergillosis but was excluded from the present study due to a concurrent systemic disease (Leishmaniasis), which may have affected the nasal mucosa and influenced treatment outcomes in SNA. (**C**) Plant foreign body removed from the nasal cavity (granule).

Diagnosis of an additional nasal cavity pathology was as follows (Figure 6): A nasal plant foreign body was diagnosed based on endoscopic findings, as CT did not provide meaningful information in this case. Conversely, bony changes—such as those resulting from dental root pathology or fractures—were detectable on CT but not visible on rhinoscopy.

### 3.4. Cross-Sectional Imaging

In all dogs, CT or MRI (performed in 29/30 dogs) revealed unilateral turbinate destruction and fluid accumulation in one nasal cavity. In two dogs, increased secretions were also noted in the contralateral nasal cavity. An accumulation of soft tissue and fluid isodense material in the frontal sinus was detected in follow-up examinations in 17/29 dogs (59%; for 1 sA dog, no cross-sectional imaging was available). Of these 17 dogs, 15 dogs (88%) belonged to group pA. In 13/15 pA dogs, sinusitis was detected at the first presentation, including 4 dogs with mild fluid accumulation in the frontal sinus who were ultimately diagnosed with SNA at their second presentation. In 2/15 pA-dogs, sinusitis developed during the disease, despite treatment.

Frontal sinus involvement was observed in 2/17 dogs (12%) in group sA. One of these dogs had evidence of old fractures in the frontal sinus area, while the other presented with periodontopathy of the left canine tooth.

Fisher’s exact test showed that dogs with primary aspergillosis (pA) were significantly more likely to have sinusitis than those with secondary aspergillosis (sA) (OR = 16.88, 95% CI: 2.70–90.02, *p* = 0.002).

In dogs from group pA with frontal sinus involvement, cribriform plate contour disruption—interpreted as lysis or decalcification—was present in 4 of 15 cases (27%).

### 3.5. Diagnostic Rhinoscopy

**Figure 7:**
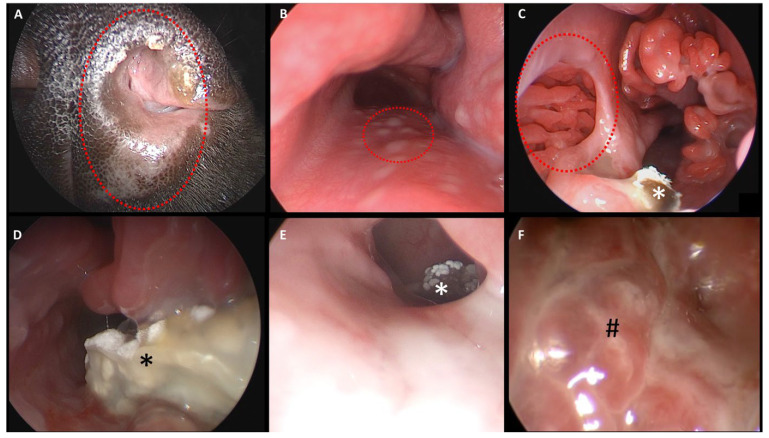
**Rhinoscopic findings in dogs with sinonasal aspergillosis (SNA).** (**A**) Depigmentation of the nasal muzzle and nasal bulb (bulbus nasi). (**B**) Lymphatic activation of the mucosa at the nasal exit showing a cobblestone appearance (dotted circle). (**C**) Septal defect (dotted circle). (**D**) Fungal granuloma within the nasal cavity. (**E**) View from the caudal nasal cavity into the pathologically widened frontal sinus containing a fungal granuloma. (**F**) Severely altered, vesicular nasal mucosa with mucosal blebs (#). (*) Indicates fungal granulomas.

Endoscopic findings (Figure 7), consistent with cross-sectional imaging (where available), showed involvement of the right nasal cavity in 13 [sA: 5]/30 dogs (43%) and of the left nasal cavity in 17 [sA: 6]/30 dogs (57%). In 2/30 dogs (7%; both from group pA), increased secretions were also observed in the contralateral nasal cavity.

*Location of the fungal granulomas:* At the time of diagnosis, fungal granulomas accompanied by purulent secretions and turbinate destruction were identified endoscopically in all dogs. The granulomas were located in the nasal cavity in 15/30 dogs (50%) and in the frontal sinus in 15/30 dogs (50%; 13 pA and 2 sA; please note that in 2 dogs, the frontal sinus involvement was not associated with the presence of a fungal granuloma).

*Frontal sinus granuloma and degree of opening of the frontal sinus into the nasal cavity:* In 2/15 dogs (13%) with a fungal granuloma located in the frontal sinus, information regarding the width of the frontal sinus opening into the nasal cavity was not available. In 8/15 dogs (53%; 7 pA and 1 sA), direct endoscopic visualization and access to the frontal sinus were possible due to a pathologically widened frontal sinus opening. In 5/15 dogs (33%; 4 pA and 1 sA), the frontal sinus opening was not widened and was therefore inaccessible without endoscopic therapeutic intervention. In these five dogs, including one sA dog with periodontopathy and a hidden granuloma in the frontal sinus, the fungal granuloma was only detected after endonasal endoscopic trephination of the bony border of the frontal sinus (Figure 2 and Figure 7). No complications, such as major bleeding, were observed during or after the procedure of endonasal endoscopic trephination.

### 3.6. Therapy

#### 3.6.1. Endoscopic Therapeutic Intervention and Topical Antifungal Therapy

*Group sA:* After diagnostic rhinoscopy, the basic therapy consisted of treating the underlying disease. In 6/11 dogs, the plant foreign bodies were removed, and in 4/11 dogs with dental root disease, the affected tooth was extracted. In the dog with the frontal bone fracture, only the antifungal treatment of aspergillus infection was performed. All dogs of group sA underwent thorough endoscopic interventional debridement of the nasal cavity and, in cases of sinus involvement, of the paranasal sinuses. Access to the frontal sinuses was achieved either through pathologically dilated openings or via an endonasally created opening (by endoscopic intervention). Antifungal treatment itself was heterogenous, as described previously, and was administered at the discretion of the treating veterinarian (G.U.O. or S.R.).

*Group sA without frontal sinus involvement:* Immediately after endoscopic interventional debridement of the nasal cavity, two out of nine dogs received no further antifungal therapy. Five of the nine were treated with 1% clotrimazole cream (c-cream) only, and two of the nine dogs received irrigation treatment with clotrimazole solution (c-solution; one out of nine) or a combination of enilconazole-containing solution and c-cream after irrigation (e-solution + c-cream; one out of nine).

*Group sA with frontal sinus involvement:* Among the eleven dogs with sA, two showed frontal sinus involvement, with one presenting an impression fracture of the frontal bone and the other high-grade dental pathology. After thorough endonasal endoscopic interventional debridement of the nasal cavity and affected paranasal sinuses, one was treated with c-cream, and the other with c-solution plus c-cream.

*Group pA:* All dogs in group pA underwent thorough endonasal endoscopic interventional debridement of the nasal cavity and affected paranasal sinuses. Of the 4/19 dogs without sinus involvement, 2 were treated with just c-cream, 1 with e-solution, and 1 with e-solution + c-cream. Among the 15/19 dogs with sinus involvement, topical antifungal treatment included c-solution (3/15), c-solution followed by c-cream (2/15), e-solution followed by c-cream (4/15), or c-cream alone (6/15).

*Both groups:* After antifungal irrigation treatment or the topical application of c-cream, no difficulties in recovery from anesthesia or swelling of the upper airways were observed in any of the dogs. In accordance with the literature [26], no neurologic abnormalities were noted, even in dogs with suspected cribriform plate lysis seen on CT. According to owner reports, after irrigation with clotrimazole solution, a bloody white discharge was observed in all dogs for 1–5 days. In contrast, after flushing with enilconazole solution and the application of c-cream, only mild serous white discharge was noted for 1–3 days.

#### 3.6.2. Follow-Up Examinations and Classification of Treatment Outcomes According to Need for Further Therapy and Intervention

Two of nineteen dogs in group pA were euthanized (3 days and 4 weeks after initial treatment) due to disease severity or progression at the owner’s request. Of the remaining 17/19 dogs, 16/19 (84%) were re-presented for follow-up examinations under anesthesia, including CT and endoscopy. One dog (without sinusitis in initial diagnostics) was not re-presented due to a good clinical condition and absence of clinical signs. This dog was categorized as “zero follow-ups” (comparable to group sA) and was ultimately lost to follow-up after one year.

In the group sA, 7/11 dogs (64%) were re-presented for follow-up examinations, while 4/11 dogs were not, due to persistent absence of clinical signs (classified as “zero follow-ups”). All these dogs had no frontal sinus involvement on first diagnostics.

**Figure 8:**
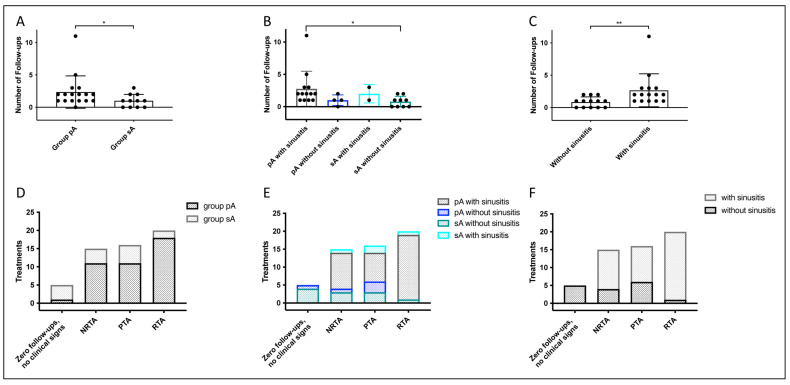
**Follow-up examinations in dogs with secondary aspergillosis (group sA) and primary aspergillosis (group pA).** (**A**) The number of follow-up examinations in dogs from group pA (16/19; 84%) was significantly higher than in group sA (7/11, 64%; * *p* = 0.04). (**B**) Number of follow-up examinations in relation to frontal sinus involvement (sinusitis) in both groups. Dogs with primary aspergillosis and sinusitis (including those diagnosed at the first follow-up examination, i.e., point of diagnosis; 15/19; 2 dogs were euthanized, and 13 dogs were rechecked) were presented significantly more often (median 2 [IQR 1–3]) than dogs from group sA without sinusitis (9/11; median 1 [IQR 0–1.5]; did not pass normality tests (alpha = 0.05); Kruskal–Wallis’ test with Dunn’s multiple comparisons; * *p* = 0.03, the corresponding rank-biserial correlation was r = 0.60, indicating a large effect size). (**C**) Comparison of follow-up examination frequency based solely on sinus involvement. Dogs with sinus involvement were presented significantly more often (median 2 [IQR 1–3]) than those without (median 1 [IQR 0–1.5]; did not pass normality tests (alpha = 0.05); Mann–Whitney’s test, two-tailed; ** *p* = 0.002, Hodges–Lehmann’s test −1.0 (95.36% CI: −2.0 to 0.0)). (**D**) Therapeutic measures during follow-up and follow-up examinations in dogs with primary aspergillosis (group pA) and secondary aspergillosis (group sA); statistically significant difference (Fisher’s exact test, *p* = 0.02)). (**E**) Therapeutic measures according to frontal sinus involvement (sinusitis) in both groups (Fisher’s exact test, *p* = 0.002). (**F**) Therapeutic measures based solely on frontal sinus involvement (Fisher’s exact test, *p* < 0.001). Zero follow-ups = no follow-up examinations under anesthesia, based on the owners’ request, in dogs that showed a complete absence of clinical signs over the long term (i.e., at least one year). In follow-up examinations under anesthesia: NRTA = no repeated antifungal therapy for aspergillosis, PTA = preventive antifungal therapy for aspergillosis, RTA = repeated antifungal therapy for aspergillosis due to refractory disease. * and ** indicate statistically significant differences, with * indicating *p* ≤ 0.05 and ** indicating *p* ≤ 0.01.

Dogs from group pA were presented significantly more often for follow-ups (median 2 [IQR 1–2.5]; 40 total follow-up examinations in 17 of 19 pA dogs) compared to group sA (median 1 [IQR 0–2]; 11 total follow-up examinations in 7 of 11 sA dogs; data did not pass normality tests (alpha = 0.05); Mann–Whitney’s test, two-tailed, *p* = 0.04, Hodges–Lehmann’s test −1.0 (95.31% CI: −2.0 to 0.0); Figure 8A). Dogs from group pA with frontal sinus involvement (15/19; 2 dogs euthanized, 13 rechecked) were presented significantly more frequently (median 2 [IQR 1–3]) than dogs from group sA with only nasal disease (9/11; median 1 [IQR 0–1.5]; did not pass normality tests (alpha = 0.05); Kruskal–Wallis’ test and Dunn’s multiple comparison test, *p* = 0.03, the corresponding rank-biserial correlation was r = 0.60, indicating a large effect size; Figure 8B).

Dogs with frontal sinus involvement (sinusitis), regardless of group, were presented significantly more frequently (median 2 [IQR 1–3]) than dogs without frontal sinus involvement (median 1 [IQR 0–1.5]; data did not pass normality tests (alpha = 0.05); Mann–Whitney’s test, two-tailed, *p* = 0.002; Hodges–Lehmann’s test −1.0 (95.36% CI: −2.0 to 0.0); Figure 8C).

There was no dog that showed clear clinical signs and nasal discharge without concurrent evidence of fungal growth on rhinoscopy or positive test results. Therefore, the applied treatment and outcome categories were appropriate for all included cases.

In follow-up examinations, dogs with pA generally required more frequent irrigation treatments or topical antifungal therapy due to recurrence or persistence of fungal infection. Refractory disease requiring repeated treatment (RTA) was observed in 18/40 follow-up examinations (45%) of pA dogs, compared to only 2 out of 11 sA dogs (18.2%) (Figure 8D). Within the pA group, in contrast to dogs with pA and sinusitis (*n* = 13), none of the four pA-dogs without frontal sinus involvement (4/19) required additional antifungal treatment during follow-up due to refractory aspergillosis (no RAT therapy; Figure 8E).

During follow-up examination of sA dogs without sinusitis, two out of nine dogs required no further topical antifungal therapy (NRTA); one of these was rechecked twice without treatment, resulting in the three NRTA entries in Figure 8E. Three out of nine sA dogs without sinusitis received preventive therapy (PTA; one dog in follow-up after treatment of refractory disease). Only one dog required repeated treatment due to refractory infection (RTA: enilconazole + c-cream). Therefore, only two dogs (one NRTA and one RAT) were rechecked twice.

In contrast, both dogs with sA and sinus involvement were examined one and three times, respectively. The dog with high-grade periodontal disease and sinusitis required three follow-up treatments due to ongoing clinical signs.

When comparing treatment frequency, (a) dogs without clinical signs (including zero follow-ups, no therapy in follow-up (NRTA), or only preventive therapy (PTA)) and (b) dogs with clinical signs due to refractory aspergillosis (RAT), treatment was significantly different in group pA (17/19 dogs: RTA *n* = 18, PTA *n* = 11, NRTA *n* = 11 and no follow-up/no control/no signs on a long-term basis *n* = 1) and sA (7/11 dogs: RTA *n* = 2, PTA *n* = 5, NRTA *n* = 4 and no follow-up/no control/no signs on a long-term basis *n* = 4; Fisher’s exact test, *p* = 0.02). In the comparison between group pA and group sA with sinusitis within each group (pA + sinusitis *n* = 13: RTA *n* = 18, PTA 8 NRTA *n* = 10; sA + sinusitis *n* = 2: RTA *n* = 1, PTA *n* = 2, NRTA *n* = 1), a statistically significant difference was found (*p* = 0.002; Fisher’s exact test). When considering only frontal sinus involvement without further differentiating primary and secondary aspergillosis (sinusitis *n* = 15; without sinusitis *n* = 13) and evaluating response to treatment, a highly statistically significant difference between treatment types was detected (*p* < 0.001; Fisher’s exact test).

Among dogs with pA and sinusitis, those initially treated with c-cream alone appeared to have more follow-up visits (*n* = 5; median 3 [IQR 1.5–8]), than those who underwent irrigation therapy with c-solution or e-solution in the same initial examination prior to c-cream application (*n* = 5; mean 2 [IQR 1–2.5]; with *n* = 1 c-solution + c-cream and *n* = 4 e-solution + cream) or those treated with solution alone (*n* = 3; mean 2 [IQR 1–2]). Note: 2/15 dogs with pA and sinusitis were euthanized after the initial therapy. However, the number of follow-up examinations did not differ significantly between these treatment groups (data did not pass normality test (alpha = 0.05), Kruskal-Wallis test, for 3 groups with solution vs. solution + c-cream vs. c-cream: *p* = 0.37 and for 4 groups with solution vs. e-solution + c-cream vs. c-solution + c-cream vs. c-cream: *p* = 0.23). Similarly, when evaluating the need for RAT in the same groups, no statistically significant differences were found (data did not pass normality test (alpha = 0.05), Kruskal-Wallis test, *p* = 0.12 for 3 groups with solution vs. solution + c-cream vs. c-cream and *p* = 0.22 for 4 groups with solution vs. e-solution + c-cream vs. c-solution + c-cream vs. c-cream).

## 4. Discussion

The present retrospective study was designed to investigate whether differences exist in treatment and prognosis between dogs with solitary SNA and those with SNA accompanied by an additional nasal disease predisposing them to SNA. To this end, we characterized dogs from both SNA groups. A total of 30 dogs were included and classified into primary aspergillosis (pA, without additional nasal disease, 19/30 dogs) and secondary aspergillosis (sA, with additional nasal disease, 11/30 dogs). Although some variation in disease severity and treatment protocols was inevitable due to individual clinical presentation, we aimed to identify general trends in treatment outcomes and overall prognosis. Unlike studies relying solely on rhinoscopy [20], our diagnostic approach at both initial and follow-up presentations was comprehensive, including cross-sectional imaging (performed in all but one sA dog at initial presentation and in all dogs during follow-up examinations) as well as thorough endoscopy of the upper respiratory tract.

Our data indicate that distinguishing between pA and sA—and assessing frontal sinus involvement—is critical for prognosis and therapeutic approach. In cases of sA, less invasive therapy (e.g., topical creams rather than solution irrigations) proved effective following removal of the inciting cause and appropriate debridement. Most sA dogs achieved complete resolution, even though some did not receive topical antifungal therapy. Therefore, diagnosing additional disease—such as foreign bodies via endoscopy or bone fractures and dental diseases via cross-sectional imaging—is essential. Dogs with sA predominantly had aspergillus infections confined to the nasal cavity, providing a helpful diagnostic indicator. Frontal sinus involvement was uncommon in sA dogs but frequently observed in pA dogs, with dogs suffering from pA showing a significantly higher risk of frontal sinusitis (odds ratio = 16.88, *p* = 0.002).

Following initial treatment, owners were advised to return their dogs for at least one anesthetized follow-up, as clinical signs may not reliably reflect disease resolution [24]. Dogs in group pA were presented for a median of two follow-up examinations [IQR 1–2.5], which exceeds prior reported data suggesting that a single treatment was sufficient in 86% of cases (please note: in the cited study, follow-up rhinoscopy was performed in only 2 of 14 dogs, and the extent of frontal sinus involvement was not specified) [27]. In the present study, repeated antifungal therapy (RAT) was required at the first follow-up examination in eight pA dogs (in total, RAT was indicated in 18 follow-up visits), whereas only one sA dog without sinusitis required RAT at the initial follow-up (in total, RAT was indicated in 2 follow-up visits). Preventive antifungal therapy was initiated in seven pA dogs at first follow-up (in 11 follow-ups in total), compared to only three sA dogs. Overall, pA dogs were presented significantly more often than those with sA (median 1 [IQR 0–2]; *p* = 0.04).

Additionally, treatment in the pA group was more “intensive”, with enilconazole or clotrimazole irrigation being used instead of “no therapy” or c-cream application, which was more typical for sA dogs. It is known that treatment with irrigations is associated with longer anesthetic sessions [4].

It is noteworthy that some dogs in the pA group continued to exhibit some kind of very mild sign of nasal cavity inflammation (e.g., very occasional sneezing or serous secretion after the owner’s use of perfume or cleaning agents). This was observed even after apparent clinical resolution and years later, as reported by owners in telephone interviews. This is consistent with the literature, which suggests that complete recovery from pA in dogs is often difficult to achieve [4]. It is presumed that these dogs may retain chronic rhinitis after mycotic infection. Relapse or a recurrence of the disease remains a possibility [20]. In contrast, owners of dogs with secondary nasal aspergillosis in this study considered their dogs to be completely cured after treatment with no signs of previous nasal cavity disease.

Several nasal diseases may predispose dogs to developing nasal aspergillus infection as a secondary mycotic infection. The resolution of both the underlying nasal disease and the aspergillus infection following the treatment of these predisposing factors in our study supports the hypothesis of primary and secondary nasal aspergillosis. In our study cohort, dogs with sA presented with additional nasal diseases, including plant foreign bodies in 6/11 dogs, previous trauma with frontal bone fracture in 1/11 dogs, and dental root pathology in 4/11 dogs. According to the literature, nasal foreign bodies are associated with an increased risk of nasal mycoses [28], as are craniofacial trauma and fractures [29]. Dental root pathologies associated with nasal aspergillosis are mainly described in humans [30].

Consistent with the literature [31], rhinoscopy was the only diagnostic modality that enabled for detection of nasal foreign bodies in 6 of 30 dogs in our study. This was critical for assigning these dogs to the group of secondary aspergillosis. Ideally, CT examination is recommended for the detection of bony fractures. Regarding dental disease, it is important to emphasize that dental root pathology, when acting as a predisposing factor for secondary aspergillosis, does not necessarily present with a clinically visible oronasal fistula [5]. Such a dental root pathology may exist without breakthrough into the nasal cavity, underscoring the importance of cross-sectional imaging or dental radiographs [5]. Considering the results of this study, especially in dogs with exclusively nasal and non-sinonasal aspergillosis, CT or dental radiography should be performed to rule out secondary aspergillosis due to dental or bony pathology.

Diagnosis of primary nasal infection with *Aspergillus* spp. in dogs presenting with nasal discharge can be challenging [4]. In this study, aspergillosis was diagnosed only at the second presentation in 4/30 dogs. In these cases, no fungal granuloma was detected during the initial evaluation, and additional tests, if performed, yielded negative results. To establish a reliable diagnosis of aspergillosis, more than one diagnostic test should ideally be conducted. Current recommendations suggest that at least two out of three diagnostic modalities yield positive results to confirm infection [5,18]. These diagnostic methods include fungal cultures, histopathological examinations, detection of fungal granulomas via rhinoscopy, and characteristic CT findings such as turbinate destruction, osteolysis, or hyperostosis. Accordingly, only dogs with a confirmed diagnosis based on more than one positive test result were included in this study.

Rhinoscopy is considered the most valuable diagnostic test in dogs with nasal disease, particularly in suspected cases of aspergillosis. It allows for direct visualization and identification of mycotic granulomas in the nasal cavity or frontal sinus, which appear as tissue-isodense material on CT scans, as demonstrated in our study population. A positive rhinoscopic finding of such granulomas is regarded as a highly specific indicator of SNA. However, a negative endoscopic finding does not definitely exclude the disease, as evidenced by the 4 out of 30 dogs diagnosed only at the second presentation. Additionally, rhinoscopy enables detection of intranasal foreign material, as described above. Therefore, endoscopic examination of the nasal cavity and paranasal sinuses is of particular importance in distinguishing between primary and secondary aspergillosis. Therapeutic interventional rhino-sinoscopy can be performed in the same anesthetic session immediately after the diagnostic exploratory rhinoscopy, allowing for simultaneous sample collection for fungal culture and histopathological analysis.

In addition to endoscopy, CT imaging contributes decisively to diagnosis [5,31]. Over the past 20 years, this modality has gained significant importance in the diagnosis of SNA [32]. However, the extent to which cross-sectional imaging techniques possess high sensitivity and specificity for the diagnosis of sinonasal aspergillosis has not been conclusively determined. In a study by Johnson et al. [11], CT findings were classified as definitely conclusive in 74% of all dogs (34/46), probably representative of nasal aspergillosis in 20% (9/46), and doubtful in 6%. In the present study, CT was essential for detecting dental root pathologies (4/30) and an old impression fracture of the frontal bone (1/30).

The cross-sectional findings in our study revealed turbinate destruction and unilateral fluid accumulation in one nasal cavity in all dogs, with increased secretions in the contralateral nasal cavity observed in 2/30 dogs (7%). Sinus involvement was noted in 17/29 dogs (59%; no cross-sectional imaging was available for one sA dog), and lysis of the cribriform plate was observed in 4/15 of pA dogs with sinus involvement (27%). The comparatively lower number of sinusitis cases in our study, compared to the literature, may be attributed to the inclusion of sA dogs. In these cases, the infection was usually confined to the nasal cavity. In contrast, other studies excluded dogs with nasal foreign bodies—i.e., those with sA—from their analyses [11]. However, when evaluating only dogs with pA in our cohort, 15 out of 19 dogs (79%) were affected by sinusitis, which is comparable to the incidence reported in the literature (78% in [33]).

Given the advantages of rhinoscopy and CT, particularly for detecting sA, this study highlights that combining both procedures is crucial and indispensable for the differential diagnosis of nasal cavity diseases in dogs. Therefore, unlike studies that evaluated dogs with SNA using rhinoscopy alone [20], both rhinoscopy and advanced imaging were employed in this study: rhinoscopy was performed in all 30 dogs, CT in 25, and MRI in 4. Only one dog with a nasal foreign body did not undergo cross-sectional imaging.

Traditionally, histopathological evidence of fungal hyphae in biopsy specimens [34,35,36] or fungal culture results were used to confirm the diagnosis [36,37]. However, both methods lack sensitivity and specificity, particularly when samples are not obtained under endoscopic guidance [2,5,11,31,38]. Our findings support this observation. Compared to other studies [39], mycological examination showed low sensitivity, both in the preliminary tests performed by referring veterinarians and in our own samples obtained during endoscopy. Therefore, the mycological examination was positive in only 12/24 dogs (50%). When fungal culture was positive in the present study, *Aspergillus fumigatus* was the predominant isolate, consistent with other studies [26,39,40].

In contrast, histopathological examination confirmed aspergillus infection in 16/27 dogs (59%), aligning with previous studies in which histopathology more frequently yielded a positive diagnosis for fungal infection than mycological examination [33]. However, discrepancies exist in the literature. For example, in a study involving dogs with visible fungal plaques, fungal cultures were positive in 59% of cases, whereas histopathological confirmation was achieved in only 47% [39].

In sA dogs, treatment of the underlying primary condition was the main therapeutic focus in this study. A distinction must be made between cases with and without sinus involvement. In nasal sA, removal of the foreign body or dental treatment, combined with a single endonasal endoscopic interventional debridement of the nasal cavity and antiseptic irrigation, was sufficient to achieve clinical resolution. Notably, two out of nine dogs with nasal sA received no antifungal therapy at all, while five out of nine dogs were treated topically with c-cream, and two out of nine dogs received an irrigation treatment with antifungal solution. Refractory aspergillosis was suspected in only one dog in this group of nasal sA. In these dogs with sA without sinusitis, four out of nine dogs with nasal sA were not followed-up due to their very good long-term clinical condition. Two out of nine dogs did not require any further topical antifungal therapy (one dog was rechecked twice without treatment; therefore, three NRTA, as shown in Figure 8E), and three out of nine dogs received preventive therapy (one dog in follow-up after refractory therapy). Only one dog had refractory infection, which required repeated treatment with enilconazole and c-cream.

In contrast to dogs with sA and nasal infestation, both dogs with sinus involvement were examined one (with preventive treatment) and three times (first examination without treatment, at the second examination (first follow-up), treatment with enilconazole and c-cream due to refractory disease, and at the third examination (second follow-up), preventive c-cream), respectively. Both dogs received repeated antifungal therapy. These cases illustrate that sA involving the sinuses presents a greater therapeutic challenge, even when the underlying cause is addressed.

In the dog with fractures of the frontal bone due to trauma, an open connection between the nasal cavity and the frontal sinus was present prior to aspergillus infection. This lack of anatomical demarcation may have triggered the sinus infection. The second dog with sinusitis had a high-grade pathology of the left maxillary canine in addition to aspergillosis. There is no evidence that the tooth pathology triggered the aspergillosis in this dog. It is also possible that both conditions occurred simultaneously and independently. This hypothesis is supported by the fact that this was the only dog with dental disease in which a fungal granuloma was identified within the frontal sinus. Furthermore, the number of follow-ups with renewed antifungal therapy was comparable to that of the dogs with primary aspergillosis.

In dogs with primary aspergillosis, the frontal sinus was most affected by the infection, as shown in 80% of dogs in this study. While the literature reports a predisposition of German Shepherds for systemic aspergillosis infections [41], it is noteworthy that in the present study, 8 out of 19 dogs (42%) in group pA belonged to retriever breeds (Labrador, Golden Retriever, and Golden Doodle). Treatment of pA can be challenging, and, unlike dogs with sA, more intense debridement and more aggressive topical antifungal drugs are often required for treatment success. In our study, antifungal therapy followed four topical treatment regimens: topical application of clotrimazole cream, irrigation with clotrimazole or enilconazole solution, and irrigation combined with c-cream. According to the literature, which usually only evaluates pA dogs [33], the dogs in the present study showed a comparable number of re-checks requiring repeated topical treatments, which was higher than in sA dogs in the present study. Interestingly, although not statistically significant, our results showed that pA dogs treated with c-cream had a higher need for follow-up examinations and repeated treatments. The clotrimazole cream in this study contained a comparable amount of clotrimazole (1%) to that used in other studies [42]. It is assumed that the increased viscosity of the cream-formulation increases the contact time of the antifungal agent with the affected mucosa, improving treatment success [20]. In a study by Vedrine and coworkers, following nasal debridement and a single application of antifungal cream, 50% of dogs with SNA were evaluated to be cured at the first follow-up endoscopy and another 40% at the second follow-up endoscopy [20].

In this study, monotherapy with c-cream was only successful in dogs diagnosed with secondary aspergillosis. However, it remains debatable whether antifungal treatment was truly necessary in these cases, as some sA dogs recovered solely through resolution of the underlying condition without any antifungal intervention. Since cream application does not significantly prolong anesthesia time, unlike irrigation therapy [43], and no adverse effects have been reported, treatment could still be justified as a precautionary measure.

Based on the results of our study, we therefore generally recommend that in dogs with pA, irrigation treatment followed by antifungal cream application should be performed after minimally invasive debridement, as previously described in the literature [27,43]. We preferred using enilconazole solution over clotrimazole solution, as it appeared to cause less nasal mucosa irritation, based on the authors’ clinical impressions and observations of nasal discharge following postprocedural flushing. This impression was further supported by owner reports describing increased nasal discharge and reduced overall well-being in dogs treated with clotrimazole compared to dogs treated with enilconazole. Other studies have also indicated that European clotrimazole formulations, which contain isopropanol and propylene glycol, may cause mucosal irritation [38]. However, further studies are needed to confirm this finding.

The study also demonstrates that the expected number of follow-up examinations and repeated treatments is higher in dogs with sinusitis than in those with disease limited to the nasal cavity. This applies to both pA and sA cases. However, it remains unclear whether primary aspergillosis, by definition, must always involve the sinuses or whether it can also be limited to the nasal cavity. Some of the dogs in this study with only nasal aspergillosis, without any detectable concurrent disease, were therefore classified as having nasal pA. Still, it is possible that due to the duration of the clinical signs (median 3 months; IQR: 1.5–6 months), potential plant-based foreign bodies had already decomposed as a result of fungal toxins and were no longer visible on rhinoscopy at the time of diagnosis. According to our findings, the clinical course in such dogs may provide further insight into whether a case represents primary or secondary aspergillosis. If a dog recovers completely following debridement (with or without antifungal treatment), secondary aspergillosis is the more likely explanation.

One limitation of the study was the variation in treatment protocols and the heterogeneous follow-up regimens, which were determined by the individual clinical needs of each dog. Despite these differing treatment regimens, meaningful conclusions can still be drawn regarding the behavior of the two disease forms (sA and pA). Another limitation is the lack of standardized diagnostic protocols. Nevertheless, neither limitation undermines the key message of this study: treatment strategies and prognoses differ between the two forms of SNA.

A further limitation is that all dogs were referred to our clinic as a secondary or tertiary care institution, and therefore, a selection bias cannot be ruled out. As a referral center with access to advanced diagnostic modalities such as CT and endoscopy and with specialization in ear, nose, and throat diseases, we may see a higher proportion of more complex cases. However, this is not unique to our institution. Other centers publishing on canine SNA are likely subject to similar referral patterns. Therefore, we believe the severity and complexity of our cases are comparable to those reported elsewhere, as reflected, for example, by the number of treatments required in dogs with primary SNA. While this may limit generalizability to first-opinion practice, it is a common limitation in the current literature on SNA. Additionally, the retrospective study design and the relatively small sample size may influence interpretation.

In conclusion, to accurately predict prognosis and to select the most appropriate antifungal therapy, it is crucial to identify secondary pathologies of the nasal cavity using CT and rhinoscopy in dogs with nasal aspergillosis. Differentiating between primary and secondary aspergillosis is essential. Secondary aspergillosis is typically confined to the nasal cavity and associated with underlying sinonasal or perinasal conditions. In most cases, treatment of the underlying cause, possibly combined with a single topical antifungal application, leads to complete recovery. In contrast, primary aspergillosis often involves the frontal sinus and requires more aggressive management, including thorough endonasal debridement, irrigation, and antifungal instillation.

## Figures and Tables

**Table 1:** **Overview of the endoscopes used in this study.**

Region	Rigid Endoscopes (Karl Storz, Tuttlingen, Germany)
Oral cavity, Oropharynx, Laryngopharynx	HOPKINS straight telescope: 10 mm; 0° or 4 mm; 0°, 30°
Nasal entrance, Nasal cavity, Paranasal sinuses	HOPKINS straight telescope: 2.7 mm; 0°, 30°, 45° or 4.0 mm; 0°, 30°
Nasopharynx, Meatus nasopharyngeus	HOPKINS II retrograde telescope: 4 mm; 120° HOPKINS straight telescope: 2.7 mm; 0°

## Data Availability

The datasets used during the current study are available from the corresponding author on reasonable request.

## Abbreviations

C	clotrimazole
C-cream	clotrimazole cream
C-solution	clotrimazole solution
CT	computed tomography
E-solution	enilconazole solution
ENT	ear, nose, and throat
FB	foreign bodies
Group pA	group primary aspergillosis
Group sA	group secondary aspergillosis
IR	idiopathic rhinitis
M1	maxillary first molar (Triadan 109/209)
M2	maxillary second molar (Triadan 110/210)
ND	nasal discharge
NRTA	no repeated antifungal therapy for aspergillosis
NP	nasal cavity pathology
OND	oronasal defects; connections between the oral and nasal cavities, such as cleft palate, periodontal pathologies, oronasal fistulas
P2	maxillary second premolar (Triadan 106/206)
P3	maxillary third premolar (Triadan 107/207)
P4	maxillary fourth premolar (Triadan 108/208)
PTA	preventive antifungal therapy for aspergillosis
RTA	repeated antifungal therapy for aspergillosis
SNA	sinonasal aspergillosis
